# Anterior Cruciate Ligament Reconstruction Does Not Impact Career Earnings After Return to Play in National Basketball Association Athletes

**DOI:** 10.1016/j.asmr.2021.07.010

**Published:** 2021-08-21

**Authors:** Muhammad J. Abbas, Lafi S. Khalil, Tahsin Rahman, Leena Abbas, Noel O. Akioyamen, Brendan J. Farley, Talal Bazzi, Kelechi R. Okoroha

**Affiliations:** a Department of Orthopaedic Surgery, Henry Ford Hospital, Detroit, Michigan, U.S.A.; b University of Illinois at Chicago College of Medicine, Chicago, Illinois, U.S.A.; c Wayne State University School of Medicine, Detroit, Michigan, U.S.A.; d Central Michigan University College of Medicine, Mount Pleasant, Michigan, U.S.A.; e Department of Orthopedic Surgery, Mayo Clinic, Minneapolis, Minnesota, U.S.A.

## Abstract

**Purpose:**

To quantify the financial impact of an anterior cruciate ligament (ACL) injury on the remaining career earnings of National Basketball Association (NBA) players.

**Methods:**

We performed a retrospective review of all NBA players who had an ACL rupture between 2000 and 2019. Players were matched to healthy controls by age, position, body mass index, and player efficiency rating at the time of injury (index year). Player information collected included demographic information, position, team role, draft pick, date of injury, contract length, and earnings during the 3 years before and 7 years after the index year, as well as new contract length and earnings after injury.

**Results:**

A total of 12 players (22%) did not return to play (RTP). No statistically significant difference in annual earnings was present at any time point between cohorts. When we examined the mean difference in earnings between the first 3 post-index seasons and the 3 pre-index seasons, both the ACL and control cohorts showed increased salaries as players’ careers progressed, without a significant difference in earnings. When comparing cohorts, we found no significant difference in the length and earnings of contracts during the index year. Furthermore, there was no significant difference in the length or earnings of the first new contract signed after the index year between cohorts. Additionally, NBA players who were able to RTP after ACL reconstruction were more likely to experience increased earnings if they had greater experience and performance prior to their injury (*P* < .01).

**Conclusions:**

Our study found that NBA players did not experience diminished earnings after RTP from an ACL reconstruction when compared with matched controls. Furthermore, no differences were seen in lengths of new contracts or in contract earnings between cohorts. Players with greater experience and performance prior to injury were more likely to have increased earnings after ACL reconstruction.

**Level of Evidence:**

Level III, retrospective case-control study.

Anterior cruciate ligament (ACL) ruptures are common and potentially career-altering injuries in National Basketball Association (NBA) athletes.^1^^,^^2^ Despite surveillance modalities, the annual incidence of ACL ruptures in NBA players is estimated to be 2.54 per year, with roughly 80% to 90% of players able to return to play (RTP) in the NBA.^2^, ^3^, ^4^ The average timing of RTP after ACL reconstruction in NBA players is approximately 11.6 months (95% confidence interval, 7.5-15.7 months).^4^ Additionally, it has been shown that these athletes may have shorter careers after ACL reconstruction, which may impact their potential career earnings.^4^^,^^5^ However, recent literature has shown that NBA athletes RTP at high levels of performance after ACL reconstruction.^3^^,^^6^ These studies compared performance before and after RTP from an ACL reconstruction and showed favorable results, indicating that these athletes are not necessarily less valuable after injury.^6^^,^^7^

The value of NBA players is influenced by their in-game statistics; thereby, after an ACL injury, their perceived future value may be reflected in their future earnings and career contracts.^8^ Secrist et al.^9^ examined the economic impact of ACL reconstruction in National Football League (NFL) athletes. They found a reduction in mean earnings over a 4-year period of $2,070,521 in players who had ACL injuries when compared with controls. Additionally, they found that ACL-injured NFL players remained in the league at a lower percentage than did healthy control players for each subsequent season after the index year. Although the financial implications of an ACL injury have been evaluated in NFL athletes, there continues to be a paucity of literature delineating the potential impact of ACL reconstruction imposed on NBA athletes’ career earnings.

In light of the aforementioned literature, it is clear that RTP rates and performance in NBA players after ACL reconstruction have improved over the decades. However, it is unknown whether the success of ACL reconstruction in allowing athletes to return to high levels of competition is reflected in the value of the contracts these players earn. The purpose of this study was to quantify the financial impact of an ACL injury on the remaining career earnings of NBA players. We hypothesized that a significant decrease in total career earnings would be seen for players with ACL injuries after RTP compared with healthy NBA athletes.

## Methods

We performed a retrospective review of all NBA players who experienced an ACL rupture and underwent reconstruction between 2000 and 2019. Ultimately, these players were compared with a healthy, uninjured control group of NBA players to analyze the financial earnings of each group and compare them with one another in relation to the date of ACL reconstruction.

Players were identified by way of third-party publicly available resources, such as player profiles, available news reports through internet searches, injury reports, team websites, NBA.com, and basketball-reference.com, using methods similar to previous studies.^10^, ^11^, ^12^, ^13^, ^14^ All documented cases of ACL rupture were verified using 2 independent sources and then cross-referenced with player statistics to confirm absence from game play during this period. Any NBA player with a history of ACL rupture from 2000 through 2019 was included from the initial search. Players were excluded if their ACL injury was associated with significant concomitant injuries, such as multiligamentous knee injuries, or other lower-extremity injuries; however, concomitant meniscal injuries were seldom reported in public sources, and therefore, players with such injuries were not excluded if present. Players were also excluded if they were injured outside of NBA play, such as while participating in outside leagues, or if they had a history of ACL rupture, a history of surgery on either leg prior to injury, or multiligament injuries. Those players with ACL injuries sustained during the recent 2019-2020 season were excluded because of a lack of opportunity to RTP prior to study initiation.

The season or off-season during which an NBA player sustained an ACL rupture was set as the index year for the purposes of data collection. Player information was collected in the index season, including player demographic information (e.g., age at injury, height, weight, and body mass index [BMI]), position, draft pick, and date of index year (injury year). Player efficiency rating (PER), contract length, and salary information were also collected for each player, from 3 seasons prior to the index year through 7 seasons after RTP. The initial contract and earnings at the time of injury, as well as the first subsequent contract after RTP, were included. PER is a standardized performance metric specific to the NBA that incorporates common in-game statistics (points, field goals, assists, rebounds, steals, and blocks) and is commonly used as an outcome measure in the literature.^1^^,^^2^^,^^5^^,^^10^

Players in the ACL reconstruction cohort were matched 1:2 to healthy controls by age, position, height, weight, BMI, position played, seasons played before and after the index year, total seasons played, and PER. However, on the basis of control criteria, it was not possible to find more than 1 healthy control for 14 athletes. The control group underwent collection of the same demographic, performance, and financial contract information.

### Statistical Analysis

Statistical analysis was conducted to compare players who underwent ACL reconstruction with control players. Annual earnings were evaluated between cohorts at every time point collected, from 3 years prior to the index season through 7 years after RTP. Initial contract length, total and average contract earnings, and subsequent contract acquired were compared between groups. Additionally, the subsequent contract acquired was compared with the initial contract during the time of injury, with change in contract earnings compared between groups. The influence of PER, age, position, and draft pick for the ACL and control groups was taken into consideration by using multivariate regression analysis to evaluate these variables’ impact on player earnings.

All continuous data are reported as mean ± standard deviation, whereas categorical data are reported as counts and column percentages. For continuous variables, univariate 2-group comparisons were performed using the independent 2-sample *t* test if the variable was normally distributed and using the Wilcoxon rank sum test if the variable was non-normally distributed. For categorical variables, univariate 2-group comparisons were performed using the χ^2^ test when expected cell counts were greater than 5 and using the Fisher exact test when expected cell counts were 5 or less. Repeated measures were performed to determine whether players’ earnings changed differently over time between groups. For repeated-measures analyses, data are reported as adjusted means (standard errors [SEs]). Mixed-effects models were fit using maximum likelihood methods to see how certain characteristics contributed to player earnings. Statistical significance was set at *P* < .05. All analyses were performed using SAS software (version 9.4; SAS Institute, Cary, NC).

## Results

### Demographic Characteristics

Fifty-four players who had an ACL rupture were included in the final analysis. One player was excluded from the final analysis because he had a multiligament injury. A total of 12 players (22%) either did not RTP, played fewer than 15 games in the season after RTP, or played fewer than 40 games in the first 2 seasons after RTP. There was no statistically significant difference in age, BMI, PER, position played, or number of seasons played between the ACL and control cohorts (Table 1). Players in the ACL cohort were significantly more likely to have been an earlier draft pick than players in the control cohort.

**Table 1:** Demographic Characteristics of NBA Players Sustaining ACL Rupture Versus Matched Controls

Characteristic	ACL (n = 54)	Control (n = 94)	*P* Value
PER	12.89 ± 5.70	13.05 ± 5.07	.86
Age, yr	24.94 ± 3.93	24.46 ± 3.41	.43
Height, in	78.93 ± 3.75	78.87 ± 3.51	.931
Weight, lb	221.13 ± 28.11	221.38 ± 27.21	.957
BMI	24.89 ± 1.96	24.93 ± 1.77	.91
Position			
Point guard	14 (26)	24 (26)	.982
Shooting guard	11 (20)	21 (22)	
Small forward	5 (9)	10 (11)	
Power forward	14 (26)	25 (27)	
Center	10 (19)	14 (15)	
Draft pick	2.30 ± 2.12	12.94 ± 8.09	<.01∗
Seasons before injury	3.50 ± 3.47	3.26 ± 3.07	.792
Seasons after injury	4.94 ± 4.08	4.36 ± 3.51	.491
Total seasons played	8.98 ± 4.60	8.61 ± 4.53	.63
RTP			
Yes	42 (78)		
No	12 (22)		

NOTE. Continuous variables are presented as mean ± standard deviation, and categorical variables are presented as frequency (percentage).

BMI, body mass index; NBA, National Basketball Association; PER, player efficiency rating; RTP, return to play.

∗ Statistically significant (*P* < .05).

### Earnings

**Fig 1:**
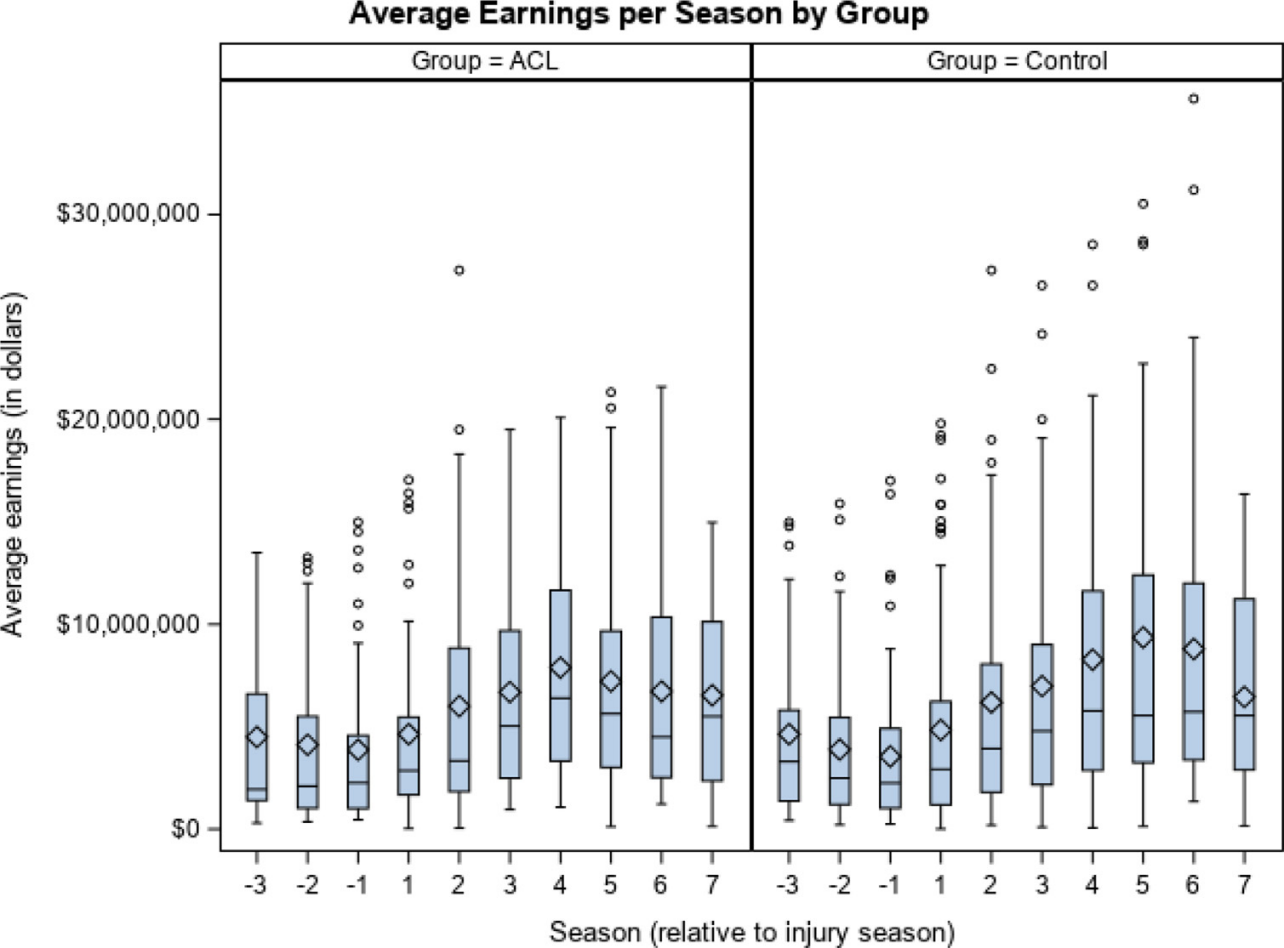
Average earnings per season by group. Pre-index seasons are denoted by seasons –1, –2, and –3. Post-index seasons are denoted by seasons 1 through 7. The index season (season 0) is omitted from this figure. There are no significant differences in average earnings (*P* < .05) among any seasons. (ACL, anterior cruciate ligament.)

When evaluating the annual earnings of NBA players with a history of ACL rupture, we found no statistically significant difference at any time point when compared with matched controls (Fig 1). When we examined the mean difference in earnings between the first 3 post-index seasons and the 3 pre-index seasons, both the ACL and control cohorts showed increased salaries as their careers progressed, with no significant difference present in earnings reported ($1,662,574 ± $4,685,411 vs $2,593,673 ± $5,323,361, *P* = .79). When earnings were examined based on playing position, no significant differences were observed between the control and ACL cohorts for guards, forwards, or centers (Table 2).

**Table 2:** Earnings Comparison by Player Position for Post-index Seasons 1 Through 3 Versus Pre-index Seasons 1 Through 3

	ACL	Control	*P* Value
Guards	n = 25	n = 45	
Average earnings in pre-index seasons, US $	4,373,695 ± 4,470,460	3,893,989 ± 3,972,354	.626
Average earnings in post-index seasons, US $	5,145,713 ± 5,522,308	5,652,051 ± 6,064,780	.943
Mean difference, US $	1,662,574 ± 4,685,411	2,593,673 ± 5,323,361	.785
Forwards	n = 19	n = 35	
Average earnings in pre-index seasons, US $	3,469,015 ± 3,393,684	2,751,200 ± 2,978,758	.551
Average earnings in post-index seasons, US $	6,222,878 ± 5,149,565	5,057,099 ± 4,895,942	.337
Mean difference, US $	2,859,271 ± 4,094,443	3,124,148 ± 3,891,856	.925
Centers	n = 9	n = 14	
Average earnings in pre-index seasons, US $	1,843,958 ± 1,098,476	3,083,083 ± 1,515,511	.081
Average earnings in post-index seasons, US $	4,156,448 ± 3,990,242	6,624,004 ± 5,371,390	.27
Mean difference, US $	2,944,089 ± 3,506,774	5,066,223 ± 4,863,152	.342

NOTE. Continuous variables are presented as mean ± standard deviation.

ACL, anterior cruciate ligament.

Regression models were used to assess the impact of player characteristics on career earnings. For every additional season of experience a player had prior to an injury, players saw a 14.00% increase in earnings (β estimate, 0.13 [SE, 0.03]; *P* < .01). Additionally, for every point increase in the mean PER of pre-index seasons 1 through 3, players saw a 10.74% increase in earnings (β estimate, 0.10 [SE, 0.02]; *P* < .01). Moreover, for every additional year older a player was in the season of injury, players saw a 4.81% increase in earnings (β estimate, 0.05 [SE, 0.02]; *P* = .04).

### Contract Information

When we examined the contract information of NBA players with ACL rupture versus healthy control players, no significant difference was found in the length and earnings of contracts during the index year (Table 3). Furthermore, no significant difference was found in the length or earnings of the first new contract signed after the index year between cohorts. Both ACL and control players experienced a diminished contract length between the index contract and subsequent contract (mean difference of –1.12 ± 1.88 seasons [*P* < .01] and –1.24 ± 1.72 seasons [*P* < .01], respectively). It should be noted that although the subsequent contract lengths were shorter, the annual earnings increased in both cohorts (mean difference of $2,212,239 ± $7,021,551 [*P* = .04] and $2,087,627 ± $6,916,776 [*P* = .01], respectively).

**Table 3:** Contract Information Comparison Between Cohorts

	ACL	Control	*P* Value
Initial contract			
Length, yr	3.47 ± 1.45	3.45 ± 1.34	.976
Total earnings, US $	17,819,358 ± 24,513,973	19,630,439 ± 23,019,386	.45
Annual earnings, US $	3,919,077 ± 4,165,338	4,867,679 ± 4,942,150	.203
New contract			
Length, yr	2.51 ± 1.64	2.32 ± 1.57	.448
Total earnings, US $	21,263,851 ± 31,159,953	24,567,954 ± 36,403,536	.757
Annual earnings, US $	6,068,152 ± 6,944,221	6,892,720 ± 7,702,364	.435

NOTE. Continuous variables are presented as mean ± standard deviation.

ACL, anterior cruciate ligament.

## Discussion

This study found that athletes did not experience diminished earnings after RTP from an ACL reconstruction. Furthermore, no differences were seen in contract lengths or contract earnings between cohorts at any time point. An interesting finding was that NBA players who RTP after ACL reconstruction were more likely to experience increased earnings if they had greater experience and performance prior to their injury, which parallels the trajectory of earnings in a typical NBA career.^8^

Professional athletes leverage their performance and value to their team to negotiate and acquire long-term, lucrative contracts. After an injury, players, coaches, and organizations are heavily invested in the recovery potential and prognosis of athletes. Recent literature has shown success in NBA athletes after ACL reconstruction, which has shifted perceptions of ACL injury from a devastating and potentially career-ending injury to a promising and encouraging recovery with appropriate surgical and rehabilitative care. As the perception of sustaining this injury loses its stigma, athletes would be expected to maintain their value.

There is a paucity of literature evaluating the impact of an ACL rupture on the earnings of NBA athletes. Secrist et al.^9^ performed a cohort study evaluating the financial implications of ACL rupture on NFL athletes. Their study found that, on average, NFL players who have ACL ruptures earn $2,070,521 less than matched controls over a 4-year period after RTP. Navarro et al.^15^ evaluated the financial impact of RTP after a concussion in NFL players. They found that athletes in the concussion cohort exhibited a mean salary reduction of $300,000 ± $1,300,000 per year. Contrary to the literature on professional football players, our study found that NBA players did not exhibit reductions in earnings after ACL reconstruction as compared with healthy controls. In comparison to NFL players, NBA players have longer average careers and contracts. NBA contracts usually also have a longer period that is guaranteed. This allows NBA players to come back comfortably after an injury without a direct impact on their pay. This may also allow a longer period to prove themselves after returning from injury to secure a subsequent contract.^16^ Additionally, the physical and demanding nature of professional football may create a perception that a player returning from injury carries a greater risk and therefore becomes less valued.^17^ It has been reported that among NFL players, ACL reinjury occurs at a rate of 25%, which is notably higher than the reported 11.8% incidence in NBA players.^10^^,^^18^ Finally, it has been found that although NBA players are reported to reach preinjury baseline performance by the second season after RTP, NFL players exhibit reduced performance for 3 seasons after RTP.^6^ These factors contribute to the retained earnings after RTP from ACL reconstruction in NBA athletes.

The ability of NBA players to achieve preinjury baseline performance after a serious injury is encouraging after ACL reconstruction. Many studies have evaluated the effects of player characteristics on the earnings of NBA players. Sigler and Compton^8^ examined 540 NBA players during the 2017-2018 season and found that a player’s number of years of experience significantly impacted his salary (regression coefficient, 644.0; *P* < .01). Furthermore, they found that performance metrics (points per game, rebounds, and assists) significantly influenced earnings (regression coefficients of 471.1 [*P* < .01], 705.1 [*P* < .01], and 360.3 [*P* = .04], respectively). Similarly, Lyons et al.^19^ found performance metrics (field goal percentage, rebounds, and assists) to be significant determinants of NBA player earnings (regression coefficients of 219,034.76 [*P* = .04], 516,373.44 [*P* < .01], and 461,354.68 [*P* = .01], respectively). In line with the prior literature, our study found that preinjury years of experience, player age, and preinjury performance significantly impacted earnings after ACL reconstruction. It stands to reason that players with greater preinjury experience and performance would earn the confidence of their organization upon their return from injury because their proficiency has been established. Although age was not evaluated in previous studies, it is expected that player age and years of NBA experience are highly correlated and, thus, the finding of increased earnings with age is an expected finding.

The performance of NBA players after ACL reconstruction has been shown to reach equivalent levels to healthy controls and to preinjury baseline at long-term follow-up.^4^, ^5^, ^6^, ^7^ Busfield et al.^1^ performed a case series of NBA athletes after RTP from an ACL rupture and found that no significant reduction in PER existed after RTP when compared with healthy controls. Mai et al.^6^ showed that NBA players returned to preinjury levels of performance after their first season after RTP, suggesting no long-term performance impact from ACL injuries. Furthermore, Harris et al.^4^ reported that career longevity after RTP was unchanged compared with healthy controls and found that, on average, players continued to play in the NBA for 5.1 ± 3.1 seasons. The ability of athletes in the NBA to perform at preinjury levels without a reduction in career longevity may explain the results of our investigation, in which their career earnings were maintained after ACL reconstruction. Moreover, we found that athletes who RTP after ACL injuries continued to see increased earnings throughout their careers in line with those of matched controls. This finding can likely be attributed to the constant increase in the league salary cap year after year.^20^ Additionally, collective bargaining agreements have allowed players to benefit from the ever-increasing earnings reported by the league.^21^ It should be noted that a number of intangible factors may play a role in an athlete’s ability to RTP, such as the psychological factor of fear of reinjury, which may limit earnings potential after ACL reconstruction.^22^ However, for the players who are able to successfully RTP, this study highlights that greater experience as well as performance preceding injury is a significant determinant of earnings potential after ACL reconstruction, showing that the value of athletes depends more on their proven contribution on the court and is not diminished by an ACL injury, as compared with healthy controls.

### Limitations

This study is not without limitations. Although data acquisition using publicly available resources has been performed in prior studies, these resources lack access to official medical records and the official NBA injury database.^23^ As such, it is not possible to gain access to information regarding concomitant injuries or injury classification. An additional limitation is that all earnings and contract information was collected using publicly available data, and we did not have access to firsthand contracts to verify the data or consider additional contract incentives and bonuses. Of note, it is possible that players included in the final analysis sustained injuries prior to their professional careers, and this may confound the results of our study. Furthermore, this study was not able to account for several confounding factors involving contract negotiations among the athletes, agents, and teams. A power analysis was not performed to determine cohort sizes; rather, acquisition of data included all players over the 20-year period from 2000 through 2019. Although a 1:2 matched analysis of injured players to control players was performed to improve the power of statistical analysis, it is possible that this study was not adequately powered to determine a significant difference. Finally, initial contracts on entering the NBA may be determined by draft pick position, which was significantly different between groups. Consequently, the total earnings amount on a contract is determined by several factors such as cap space, draft pick position, length of contract, and bonuses, as well as additional factors, which may account for the large standard deviations presented in this study.

## Conclusions

Our study found that NBA players did not experience diminished earnings after RTP from an ACL reconstruction when compared with matched controls. Furthermore, no differences were seen in lengths of new contracts or in contract earnings between cohorts. Players with greater experience and performance prior to injury were more likely to have increased earnings after ACL reconstruction.

## Supplementary Data

ICMJE author disclosure forms

## References

[1] Busfield B.T., Kharrazi F.D., Starkey C., Lombardo S.J., Seegmiller J. (2009). Performance outcomes of anterior cruciate ligament reconstruction in the National Basketball Association. Arthroscopy.

[2] Minhas S.V., Kester B.S., Larkin K.E., Hsu W.K. (2016). The effect of an orthopaedic surgical procedure in the National Basketball Association. Am J Sports Med.

[3] Nwachukwu B.U., Anthony S.G., Lin K.M., Wang T., Altchek D.W., Allen A.A. (2017). Return to play and performance after anterior cruciate ligament reconstruction in the National Basketball Association: Surgeon case series and literature review. Phys Sportsmed.

[4] Harris J.D., Erickson B.J., Bach B.R. (2013). Return-to-sport and performance after anterior cruciate ligament reconstruction in National Basketball Association players. Sports Health.

[5] Kester B.S., Behery O.A., Minhas S.V., Hsu W.K. (2017). Athletic performance and career longevity following anterior cruciate ligament reconstruction in the National Basketball Association. Knee Surg Sports Traumatol Arthrosc.

[6] Mai H.T., Chun D.S., Schneider A.D. (2017). Performance-based outcomes after anterior cruciate ligament reconstruction in professional athletes differ between sports. Am J Sports Med.

[7] Mehran N., Williams P.N., Keller R.A., Khalil L.S., Lombardo S.J., Kharrazi F.D. (2016). Athletic performance at the National Basketball Association Combine after anterior cruciate ligament reconstruction. Orthop J Sports Med.

[8] Sigler K., Compton W. (2018). NBA players’ pay and performance: What counts?. Sport J.

[9] Secrist E.S., Bhat S.B., Dodson C.C. (2016). The financial and professional impact of anterior cruciate ligament injuries in National Football League athletes. Orthop J Sports Med.

[10] Khalil L.S., Jildeh T.R., Tramer J.S. (2020). Effect of Achilles tendon rupture on player performance and longevity in National Basketball Association players. Orthop J Sports Med.

[11] Jildeh T.R., Okoroha K.R., Taylor K.A. (2019). Effect of concussions on the performance of running backs and wide receivers in the National Football League. Am J Sports Med.

[12] Keller R.A., Mehran N., Khalil L.S., Ahmad C.S., ElAttrache N. (2017). Relative individual workload changes may be a risk factor for rerupture of ulnar collateral ligament reconstruction. J Shoulder Elbow Surg.

[13] Marshall N.E., Jildeh T.R., Okoroha K.R., Patel A., Moutzouros V., Makhni E.C. (2018). Implications of core and hip injuries on Major League Baseball pitchers on the disabled list. Arthroscopy.

[14] Okoroha K.R., Kadri O., Keller R.A., Marshall N., Cizmic Z., Moutzouros V. (2017). Return to play after revision anterior cruciate ligament reconstruction in National Football League players. Orthop J Sports Med.

[15] Navarro S.M., Sokunbi O.F., Haeberle H.S. (2017). Short-term outcomes following concussion in the NFL: A study of player longevity, performance, and financial loss. Orthop J Sports Med.

[16] Arthur R. The shrinking shelf life of NFL players. *The Wall Street Journal*. February 29, 2016.

[17] Edwards T., Spiteri T., Piggott B., Haff G.G., Joyce C. (2018). A narrative review of the physical demands and injury incidence in American football: Application of current knowledge and practices in workload management. Sports Med.

[18] Stuhlman C.R., Owens C.J., Samuelson E.M. (2019). Recurrent anterior cruciate ligament tears in the National Football League: A case-control study. Orthop J Sports Med.

[19] Lyons R., Jackson E.N., Livingston A. (2015). Determinants of NBA player salaries. Sport J.

[20] NBA Salary Cap History. 2020. Basketball Real GM2020. Accessed 14 December 2020, https://basketball.realgm.com/nba/info/salary_cap

[21] Gough C. National Basketball Association total league revenue from 2001/02 to 2018/19. December 12, 2020. Statistica2020. Accessed 14 December 2020, https://www.statista.com/topics/967/national-basketball-association/

[22] McCullough K.A., Phelps K.D., Spindler K.P. (2012). Return to high school- and college-level football after anterior cruciate ligament reconstruction: A Multicenter Orthopaedic Outcomes Network (MOON) cohort study. Am J Sports Med.

[23] Maak T.G., Mack C.D., Cole B.J., Herzog M.M., Difiori J., Meisel P. (2020). Sports performance and injury research: Methodologic limitations and recommendations for future improvements. Arthroscopy.

